# Platelet Rich Plasma and Orthopedics: Why, When, and How

**DOI:** 10.1155/2015/949720

**Published:** 2015-05-10

**Authors:** Mikel Sánchez, Giuseppe Filardo, Tomokazu Yoshioka

**Affiliations:** ^1^Arthroscopic Surgery Unit, Hospital Vithas San José, 01008 Vitoria-Gasteiz, Spain; ^2^Biomechanics Laboratory-II Clinic, Rizzoli Orthopaedic Institute, 40136 Bologna, Italy; ^3^Division of Regenerative Medicine for Musculoskeletal System, Department of Orthopaedics Surgery, University of Tsukuba, Tsukuba, Ibaraki 305-8575, Japan

In present practice, orthopedic and sports medicine is making a great effort in promoting the use of minimally invasive techniques aimed at arresting or slowing down the aging and degeneration of several tissues. The scientific community has been developing new areas of research that have fuelled the emergence of regenerative medicine and tissue engineering bringing into the spotlight treatments such as those based on stem cells and platelet rich plasma (PRP). Growing interest in PRP is evident. Since PRP therapies began to be used regularly in the musculoskeletal system at the onset of this decade, this topic has now reached over 7000 publications in the scientific literature. When delving into the published research studies and reviews, two overwhelming facts stand out over the rest: a maze of clinical indications based on widely varied and even controversial results and a relative difficulty in establishing a mechanistic cause-effect relationship, facts that are fueling a rather misleading controversy.

The root of PRP controversy lies in several inconsistencies including a lack of standardization in obtaining PRP as an autologous product. PRP contains by definition a platelet concentration superior to peripheral blood. In recent years, a plethora of platelet concentration systems has been commercialized, and these are marketed under different names and acronyms but they are all sold under the umbrella term “PRP.” The final biological products are very distinct in terms of volume, color, platelet count, presence or absence of leukocytes, and unknown protein content; and the activation method, with either bovine thrombin or calcium chloride, adds more confusion to this biologic ceremony. The question we must ask is whether PRP is a homogeneous consistent biological product. Not only is it important to have a fully characterized and reproducible product, it is crucial to know how to use it as well. We have to remember that this is not a magical bullet. We should know the possible indications for its use and the volume to be applied, the frequency of application, number of applications, and so forth. PRP application for an acute injury like a bone fracture is different from treating a chronic injury such as a tendinopathy and different again from approaching a degenerative problem such as osteoarthritis. This lack of consensus is the reason for the title of this special issue.

Studies presented in this special issue cover topics ranging from preclinical to clinical research of PRP technology in different tissues. I. Giusti and I. Andia conducted two* in vitro* studies about PRP and tendon cells. The former evaluated the tenocyte cellular behavior with different platelet concentrations; I. Andia et al. analyzed the effect of uric acid in tendon cells response to PRP. Continuing the tendon pathology, D. U. Jeong et al. reviewed the clinical effectiveness of PRP in the patellar tendinopathy, and P. Randelli et al. addressed the treatment of rotator cuff injuries with PRP as well as stem cells. A. Roffi and A. Marmotti focused on PRP application for cartilage and its pathologies. The former studied the effect of PRP cryopreservation on its quality and its action over chondrocytes and synoviocytes. The latter updated the clinical knowledge of PRP in chondral surgery and in the treatment of cartilage degenerative processes. In this special issue, there are also three articles related to knee pathology. J. Zellner et al. carried out both* in vitro* and* in vivo* assays in order to study the effect of PRP and BMP7 in meniscal defects. L. Andriolo et al. presented a systematic review about biological effects of PRP in the reconstruction of the anterior cruciate ligament. In addition, M. Sanchez et al. showed a series of PRP application guidelines for several knee surgeries. Finally, F. Salamanna et al. conducted an extensive literature review of preclinical studies with PRP in different musculoskeletal tissues.

As Bertolt Brecht wrote in* Life of Galileo* “The aim of the science is not to open the door to infinite wisdom but to set a limit to infinite error”, these works are only a few examples of the larger number of studies still needed to answer questions that give name to this special issue. We are at the nascent end of a new health technology and, as it happened in the era of antibiotics, we must lead to a future in which PRP should be characterized both for each patient and for each application protocol. And as it happened many times in the history of medicine, we are constantly passing through the three phases described by Schopenhauer: “All truth passes through three stages. First, it is ridiculed. Second, it is violently opposed. Third, it is accepted as being self-evident.” However, we realize that now we face the paradox of Socrates: we know that we know nothing. Therefore, further studies about PRP as presented here are necessary to keep shedding light on this topic.

*Mikel Sánchez* *Giuseppe Filardo* *Tomokazu Yoshioka*

